# Photoperiod and Light Spectrum Modulate Daily Rhythms and Expression of Genes Involved in Cell Proliferation, DNA Repair, Apoptosis and Oxidative Stress in a Seabream Embryonic Stem Cell Line

**DOI:** 10.1007/s10126-025-10418-z

**Published:** 2025-01-31

**Authors:** Alba Vergès-Castillo, Patricia Herrera-Pérez, Carlos Pendón, Águeda J. Martín-Robles, José A. Muñoz-Cueto

**Affiliations:** 1 Departamento de Biología, Facultad de Ciencias del Mar y Ambientales, Universidad de Cádiz, 11510 Puerto Real (Cádiz), Spain; 2 Área de Bioquímica y Biología Molecular, Departamento de Biomedicina, Biotecnología y Salud Pública, Facultad de Ciencias, Universidad de Cádiz, 11510 Puerto Real (Cádiz), Spain; 3 Instituto Universitario de Investigación Marina (INMAR), Campus de Excelencia Internacional del Mar (CEIMAR), The European University of the Seas (SEA-EU), 11510 Puerto Real (Cádiz), Spain; 4 Instituto de Biomoléculas (INBIO), Facultad de Ciencias, Campus de ExcelenciaInternacionalAgroalimentario (ceiA3), Universidad de Cádiz, 11510 Puerto Real (Cádiz), Spain

**Keywords:** Embryonic stem cell lines, Circadian rhythms, Cell cycle, Photoperiod, Light spectrum, Teleost fish

## Abstract

The use of cell lines as alternative models for environmental physiology studies opens a new window of possibilities and is becoming an increasingly used tool in marine research to fulfil the 3R’s rule. In this study, an embryonic monoclonal stem cell line obtained from a marine teleost (gilthead seabream, *Sparus aurata*) was employed to assess the effects of photoperiod (light/dark cycles *vs* constant dark) and light spectrum (white, blue, green, blue/green and red lights) on gene expression and rhythms of cellular markers of proliferation, DNA repair, apoptosis and cellular/oxidative stress by RT-qPCR and cosinor analyses. The results obtained revealed the optimal performance of cells under blue light (LDB), with all the genes analysed showing their highest RNA expression levels and most robust daily variations/rhythms in this condition. Under LDB, the mRNA levels of cell proliferation (*pcna*), DNA repair (*cry5*), anti-apoptotic (*bcl2*) and oxidative stress (*prdx2*) markers peaked at the day-night transition, whereas pro-apoptotic (*bax*) and cell stress (*hsp70*) markers showed their highest expression at the night-day transition, evidencing the strong synchronisation of the transcription of key genes involved in the cell cycle in this photoregime. The persistence of significant *pcna*, *cry5*, *hsp70* and *prdx2* rhythms after 3 days in constant darkness reveals the endogenous and circadian nature of these rhythms. Our results highlight the importance of implementing photoperiods with light–dark cycles of blue wavelengths when performing fish cell culture research. These results reinforce and extend our previous studies, confirming the importance of lighting conditions that mimic the natural environment for the proper development of fish embryos and larvae in aquaculture.

## Introduction

The light–dark (LD) cycle represents the most powerful abiotic factor for the initiation and synchronisation of many rhythmic processes, providing not only daily but also seasonal/annual temporal information to the organism (Falcón and Muñoz-Cueto 2024). This fact had been described in plants (Ke et al. 2018), animals (Asirim et al. 2020; Vatine et al. 2011) and cells (Dierickx et al. 2015; Geyfman et al. 2012; Umemura et al. 2014; Vergès-Castillo et al. 2021, 2024; Whitmore et al. 2000).

In aquatic environments, water acts as a chromatic filter that modulates light intensity and wavelength penetration into the water column, with blue and green wavelengths delving deeper, while UV/violet and red wavelengths are absorbed more quickly and only reach the very shallow layer (Lalli and Parsons 1997). In fish, light is absorbed by photopigments (opsins) of photoreceptor cells present in the pineal gland and retina and transduced into neural (neurotransmitters) and neurohormonal (melatonin) signals that synchronise biological rhythms in central and peripheral tissues and cells (Falcón and Muñoz-Cueto 2024; Frau et al. 2020a, b, 2022; Steindal and Whitmore 2020). But light sensitivity and entraining in fish is already present from early stages of development, before the differentiation of specialised photoreceptive structures such as the pineal gland and the retina (Davie et al. 2011; Dekens and Whitmore 2008; Martín-Robles et al. 2012). The alternation of LD cycles of appropriate wavelengths was confirmed to be very important for the optimal hatching, survival, development, growth and welfare of fish embryos and larvae, which performed better under short and medium wavelengths, i.e., blue and green lights (Villamizar et al. 2011). Moreover, in some fish species, such as the zebrafish, *Danio rerio*, light can act directly on peripheral organs and cells, which exhibit a wide variety of non-visual opsins and a light-entrainable clock (Steindal and Whitmore 2020; Vatine et al. 2009).

In previous works, we have established and characterised a monoclonal embryonic stem cell line (SAEC-H7) from morula-stage embryos of a marine teleost fish, the gilthead seabream, *Sparus aurata* (Vergès-Castillo et al. 2021), and revealed that SAEC-H7 were highly sensitive to light and exhibited an endogenous molecular clock, which is entrained by the photoperiod (Vergès-Castillo et al. 2024). In the fish embryo, light has been shown to regulate genes covering a variety of functions, such as circadian clock function, cell proliferation and apoptosis, DNA repair, pigmentation, retinal light input, melatonin synthesis and signalling, oxidative metabolism and stress response (Gavriouchkina et al. 2010; Üstündağ et al. 2019; Villamizar et al. 2011; Weger et al. 2011). However, the effects of light of different wavelengths on many of these functions remain almost unexplored in fish embryonic cells. To fill this gap, in the present study, we analysed the effects of white (LDW), blue (LDB), green (LDG), blue/green (LDBG) and red (LDR) lights, as well as constant dark conditions (DD) on daily gene expression and rhythms of cellular markers of proliferation (*pcna*), DNA repair (*cry5*), apoptosis (*bcl2*, *bax*) and stress (*hsp70*, *prdx2*) in SAEC-H7 cells by using RT-qPCR and cosinor analyses.

## Material and Methods

### Cell Culture and Routine Maintenance

The monoclonal embryonic stem cell line (SAEC-H7) obtained from morula-stage embryos of the gilthead seabream was cultured in 75 cm^2^ flasks at constant temperature (22 °C) with Leibovitz’s L-15 medium (L-15, Gibco, Waltham, MA, USA) supplemented with 10% fetal bovine serum (FBS, Gibco), 1% penicillin/streptomycin (P/S, Gibco), 1% glutamine (Gibco) and NaCl 5 M, as previously described (Vergès-Castillo et al. 2021). Cells were grown in non-illuminated incubators and the medium was renewed twice a week.

### Experimental Set-Up and Sampling

When cellular confluence was approximately 70–80%, cells were detached by using 2 ml of 0.05% trypsin (Gibco) and transferred into new 75 cm^2^ flasks. Around 2.0 × 10^5^ cells/well were seeded in 6-well plates, and after 24 h, the medium was renewed to remove debris after trypsin treatment. In order to determine the effect of different photoperiod and light spectrum regimes on cell proliferation, DNA repair, cellular apoptosis and cellular/oxidative stress, plates were maintained during 2 days under the following light regimes: LDW, 12 h white light (λ 400 to 700 nm)−12 h darkness; LDB: 12 h blue light (λ 455 nm)−12 h darkness; LDG: 12 h green light (λ 512 nm)−12 h darkness; LDBG: 12 h blue/green light (λ 455 and 512 nm)−12 h darkness; LDR: 12 h red light (λ 607 nm)−12 h darkness; DD: constant dark conditions. LED lamps were provided by Aquatic Biotechnology (Cádiz, Spain) and irradiances were properly adjusted to 150 µmol photon/m^2^s for all wavelengths employed using a QSL-2101 quantum scalar sensor (Biospherical Instrument Inc., CA, USA) placed at the bottom of the 6 well plates. In LDW, LDB, LDG, LDBG and LDR conditions, light switched on at zeitgeber time (ZT) 0 and off at ZT12. On day 3, cells were harvested every 4 h at ZT or circadian time (CT) 0, 4, 8, 12, 16, 20 and 24 during a 24 h daily cycle, collecting 6 wells at each time point (*n* = 6). Sampling during periods of darkness was performed under a dim red light.

### RNA Extraction and cDNA Synthesis

Cells were washed with PBS, detached with a cell scraper in 350 µl of Lysis Buffer RLY supplemented whit β-mercaptoethanol (Sigma-Aldrich, St Louis, Missouri, USA), and total RNA was obtained using the ISOLATE II RNA Mini kit (BIOLINE, Memphis, Tennessee, USA). Cell lysates were processed and the DNase treatment step was performed to avoid possible genomic DNA contamination, according to the manufacturer’s instructions. Total RNA was eluted in 40 μl diethylpyrocarbonate-treated water (BIOLINE, London, UK), and concentration, purity and integrity were measured in a Nanodrop (ThermoFisher Scientific, Waltham, MA, USA) and a 2100 Bioanalyzer (Agilent Technologies, Palo Alto, CA, USA). Subsequently, 500 ng of total RNA were reverse transcribed into cDNA using the iScript cDNA Synthesis Kit (Bio-Rad, Alcobendas, Spain) in a 40 μl reaction volume using a conventional T100™ Thermal Cycler system (Bio-Rad) following manufacturer’s instructions.

### Real-Time Quantitative PCR Analysis

Real-time quantitative PCR (RT-qPCR) was used to analyse the relative mRNA expression of the following target genes: cell proliferation, *proliferating cell nuclear antigen* (*pcna,* GenBank accession number KF857335.1); DNA repair, *cryptochrome-5 or 6–4 photolyase* (*cry5,* GenBank accession number XM_030415264.1); cellular apoptosis, anti-apoptotic *b-cell lymphoma-2* (*bcl2,* GenBank accession number JX975259.1) and pro-apoptotic *bcl2- associated X protein* (*bax,* GenBank accession number XM_030408214.1); cellular stress, *heat-shock protein-70* (*hsp70,* GenBank accession number EU805481.1); oxidative stress, *peroxiredoxin-2* (*prdx2,* GenBank accession number GQ252680.1). Relative expression of selected genes was analysed in a Real Time Detection System CFX96 Touch Deep Well (Bio-Rad) by triplicate. Standard curves, showing slopes close to − 3.32 and efficiencies around 100%, were generated for each set of primers using tenfold serial dilutions of cDNA (Table 1). Reactions were performed with 5 µl iTaq Universal SYBR® Green SuperMix (Bio-Rad), 0.5 µl of 10 µM forward and reverse primers (Table 1) and 4 µl of cDNA in a final volume of 10 µl per reaction, with the following amplification conditions: 95 °C for 30 s, 40 cycles of 95 °C for 5 s, annealing at 60 °C for 30 s and elongation at 72 °C for 5 s. Melting curves were also performed to ensure single amplifications and products were sequenced to confirm their identity. Non-template control and inter-run calibrator samples were used as negative and comparative controls, respectively. The 2^−ΔΔCt^ method was used to determine relative gene expression levels, using seabream *β-actin* (Genbank accession number X89920.1), and *18S* (Genbank accession number AJ291668.1) as internal control genes for normalisation (Table 1) and the LDW group (overall gene expression) and ZT0 of the LDW group (daily gene expression) as calibrators. In this way, the data are presented as the fold change in gene expression normalised to the endogenous reference genes and relative to the calibrator (Livak and Schmittgen 2001).

**Table 1:** Sequences of forward (F) and reverse (R) primers used *for proliferating cell nuclear antigen* (*pcna*), *cryptochrome-5* (*cry5*), *b-cell lymphoma-2* (*bcl2*), *bcl2- associated X protein* (*bax*), *heat-shock protein-70* (*hsp70*), *peroxiredoxin-2* (*prdx2*) and reference genes (*β-actin* and *18S*)

Name	Sequence (5′−3′)	Accession number	Amplicon size (bp)	Primer efficiency (%)
*sa_pcna_F*	AGATGAATGAACCCGTCCAG	KF857335.1	147	100
*sa_pcna_R*	CGTGTCCCATATCAGCAATC			
*sa_cry5_F*	TGAGGAGAACAATGGGAAGC	XM_030415264.1	137	105
*sa_cry5_R*	TTTCTGAACAAGGGGTCTGC			
*sa_bcl2_F*	CAGCCAGGTGCTGACATAGA	JX975259.1	118	99.9
*sa_bcl2_R*	TCAGGAGTGATGTCGAGCTG			
*sa_bax_F*	CAACAAGATGGCATCACACC	XM_030408214.1	107	98.9
*sa_bax_R*	TGAACCCGCTCGTATATGAAA			
*sa_hsp70_F*	AATGTTCTGCGCATCATCAA	EU805481.1	108	100
*sa_hsp70_R*	GCCTCCACCAAGATCAAAGA			
*sa_prdx2_F*	CAAGCAGTAAATGTGAAGGTC	GQ252680.1	99	98.9
*sa_prdx2_R*	GATTGGACGCCATGAGATAC			
*sa_βactin_F*	TCCTGCGGAATCCATGAGA	X89920.1	51	100
*sa_βactin_R*	GACGTCGCACTTCATGATGCT			
*sa_18S_F*	CGAAAGCATTTGCCAAGAAT	AJ291668.1	102	100
*sa_18S_R*	AGTTGGCACCGTTTATGGTC			

The table shows primer sequences in 5′−3′ direction, GenBank accession numbers, sizes of amplicons in base pairs (bp) and primer efficiencies (%). Primers of *pcna*, *cry5* and *18S* were own designed, those of *bcl2*, *bax*, *hsp70* and *prdx2* were selected in accordance with Morcillo et al. (2016), and *β-actin* primers were designed in accordance with Vera et al. (2013)

### Statistical and Rhythm’s Analyses

Normality and homoscedasticity assumptions were tested before the comparison of mean values by one-way ANOVA followed by Tukey’s post-hoc comparisons test, using GraphPad Prism Version 9.4.1 software (San Diego, California, USA). When required, expression values were transformed to obtain normality and homogeneity of variances. Statistically significant differences were considered when *p* < 0.05. All data were presented as mean ± standard error of the mean (SEM).

The cosinor analysis provides a cosine wave fitted by the method of the least squares and was used for the detection of significant rhythmic patterns (Refinetti et al. 2007). Rhythmicity and rhythm parameters (mesor, amplitude, acrophase and significance) of the cosinor analysis were determined with El Temps software (v. 1.228; www.el-temps.com, Professor A. Díez Noguera, University of Barcelona, Spain) and statistically significant rhythms were considered when *p* < 0.05.

## Results

To characterise the cellular outputs of SAEC-H7 in response to photoperiod and light spectrum, the overall relative expression and the daily expression rhythms profiles of genes involved in cell proliferation (*pcna*), DNA repair (*cry5*), cellular apoptosis (*bcl2* and *bax*), cellular stress (*hsp70*) and oxidative stress (*prdx2*) were analysed.

### Effects of Photoperiod and Light Spectrum on the Overall Gene Expression

**Fig. 1:**
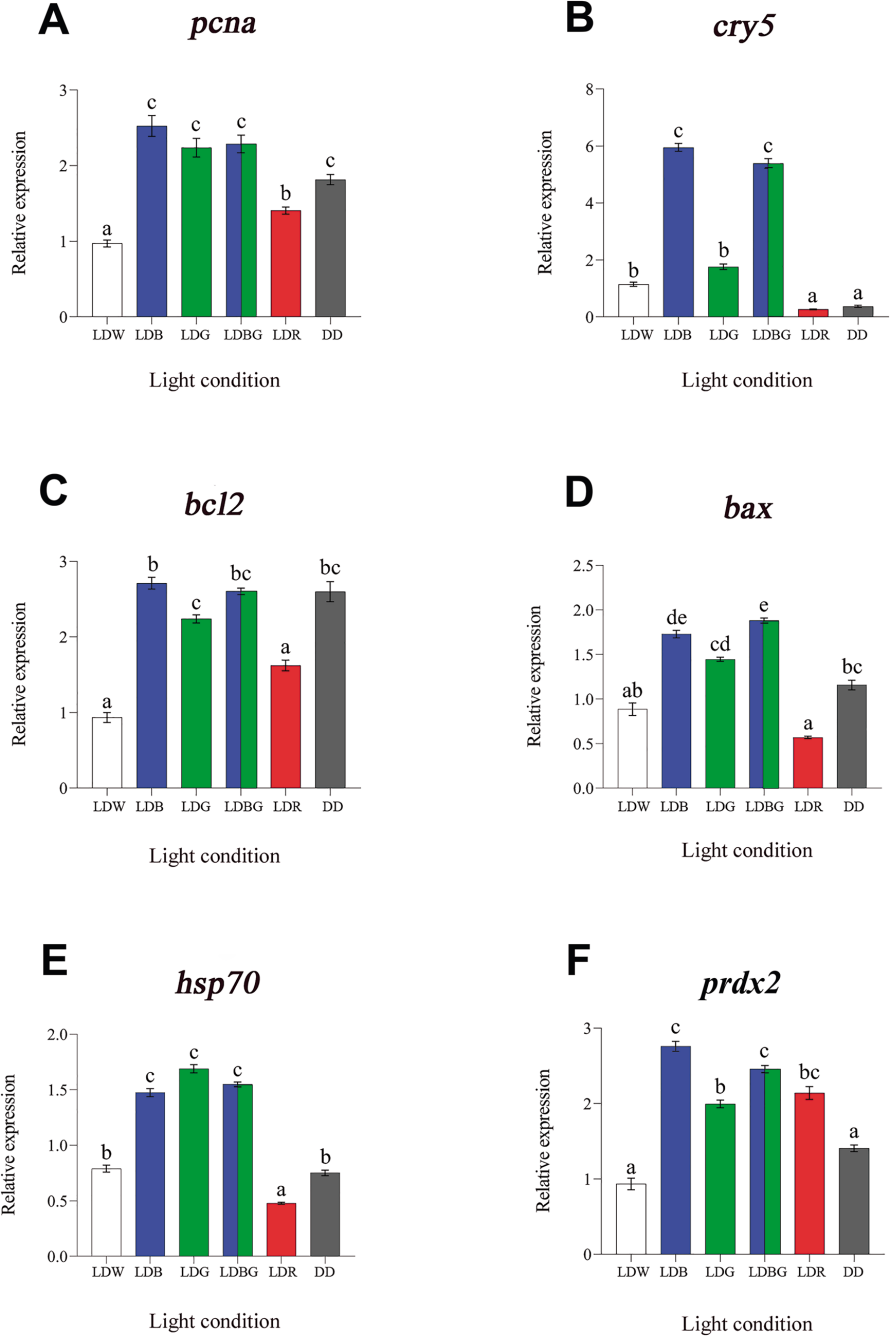
Overall relative gene expression of markers of cell proliferation (*pcna*, **A**), DNA repair (*cry5*, **B**), cellular apoptosis (*bcl2*, **C** and *bax*, **D**), cellular stress (*hsp70*, **E**) and oxidative stress (*prdx2*, **F**) in SAEC-H7 cells maintained under different photoperiods and light spectrum. Relative expression was determined by using real-time quantitative PCR (RT-qPCR). Each value represents the mean ± SEM (*n* = 42). Different letters on top of the bars indicate statistically significant differences between mean values determined by one-way ANOVA followed by Tukey’s post-hoc test (significance at *p* < 0.05 or higher). LDW: 12 h white light-12 h darkness; LDB: 12 h blue light-12 h darkness; LDG: 12 h green light-12 h darkness; LDBG: 12 h blue/green light-12 h darkness; LDR: 12 h red light-12 h darkness; DD: constant darkness

For the analysis of the overall gene expression, the average expression of ZT/CT0 to ZT/CT24 (*n* = 42) was normalised to LDW condition. The one-way ANOVA showed statistically significant differences in the relative expression of the selected genes under the different light regimes analysed (LDW, LDB, LDG, LDBG, LDR and DD). In general terms, we observed an induction of expression in almost all genes analysed in SAEC-H7 cells maintained under blue (LDB), green (LDG) and/or the combination of blue and green (LDBG) light conditions, compared to the remaining light regimes (Figs. 1 and 8).

In the case of *pcna*, the expression values were significantly higher under LDB, LDG, LDBG and constant darkness (DD), intermediate under red lights (LDR) and lower under white-light photoperiod (LDW) (Fig. 1A). Concerning the DNA repair marker *cry5*, SAEC-H7 cells showed higher transcript levels in LDB and LDBG photoregimes, three- to fivefold higher compared to LDW and LDG, and ten- to 12-fold higher in relation to LDR and DD conditions (Fig. 1B). Cell apoptosis was studied by analysing anti-apoptotic (*bcl2*) and pro-apoptotic (*bax*) gene expression (Fig. 1C and D, respectively). In both cases, there were statistical differences among LDB, LDG and LDBG mRNA levels with respect to those found in LDW and LDR, which were significantly lower. Although *bax* also displayed lower transcript levels in DD compared to the LDB and LDBG conditions (Fig. 1D), expression levels of the anti-apoptotic gene marker *bcl2* under DD were as high as those found in the LDB, LDG and LDBG conditions (Fig. 1C). Cellular (*hsp70*) and oxidative (*prdx2*) stress gene markers were also affected by photoperiod and light spectrum in SAEC-H7 cells, with expression levels being two- to threefold higher in LDB, LDG and LDBG compared to LDW and DD regimes (Fig. 1E and F). Under LDR, transcript levels were also reduced compared to LDB, LDG and LDBG for *hps70* (Fig. 1E) but not for *prdx2* (Fig. 1F).

### Effects of Photoperiod and Light Spectrum on Daily Gene Expression and Rhythms

**Fig. 2:**
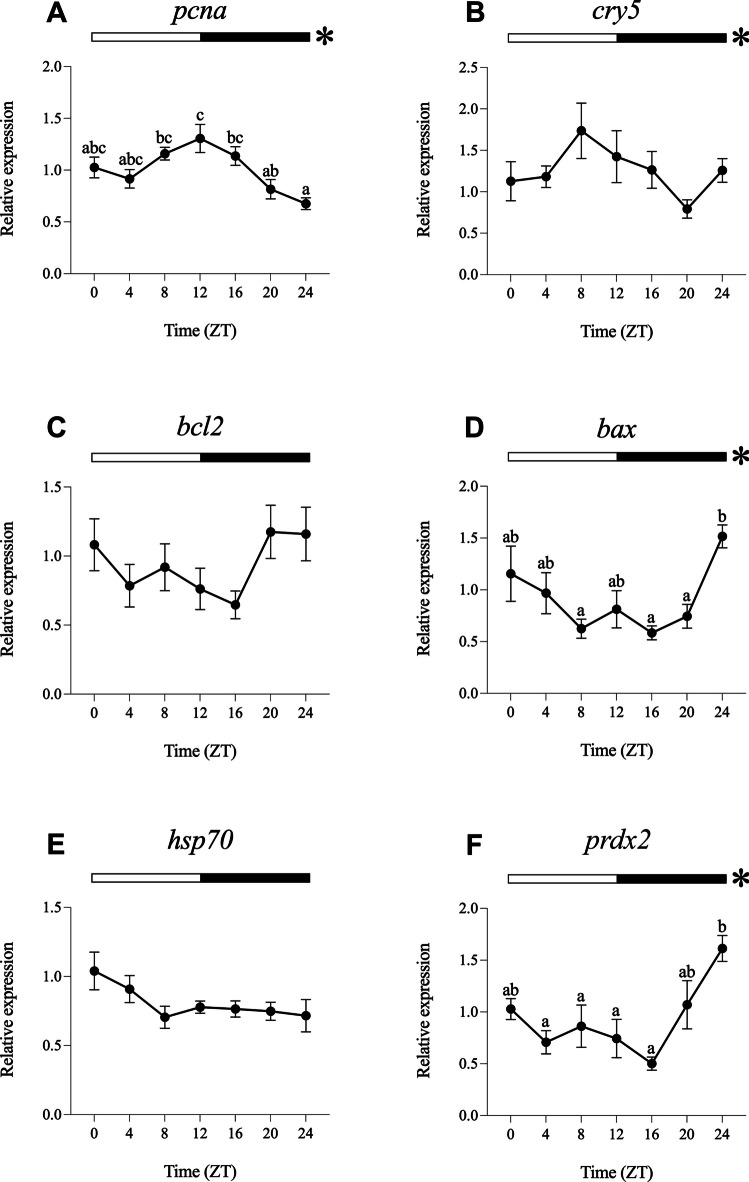
Relative daily gene expression of markers of cell proliferation (*pcna*, **A**), DNA repair (*cry5*, **B**), cellular apoptosis (*bcl2*, **C** and *bax*, **D**), cellular stress (*hsp70*, **E**) and oxidative stress (*prdx2*, **F**) in SAEC-H7 cells maintained under 12 h white light-12 h darkness photoperiod (LDW). The x-axis shows time (hours) in Zeitgeber time (ZT). Lights on was at ZT0 and lights off was at ZT12. Samples were collected every 4 h and relative expression was determined by using real-time quantitative PCR (RT-qPCR). Each value represents the mean ± SEM (*n* = 6). Horizontal bars at the top indicate light (white) and dark (black) phases. Asterisks represent significant daily rhythms determined by cosinor analysis. Different letters on top of the dots indicate statistically significant differences between mean values determined by one-way ANOVA followed by Tukey’s post-hoc test (significance at *p* < 0.05 or higher)

**Fig. 3:**
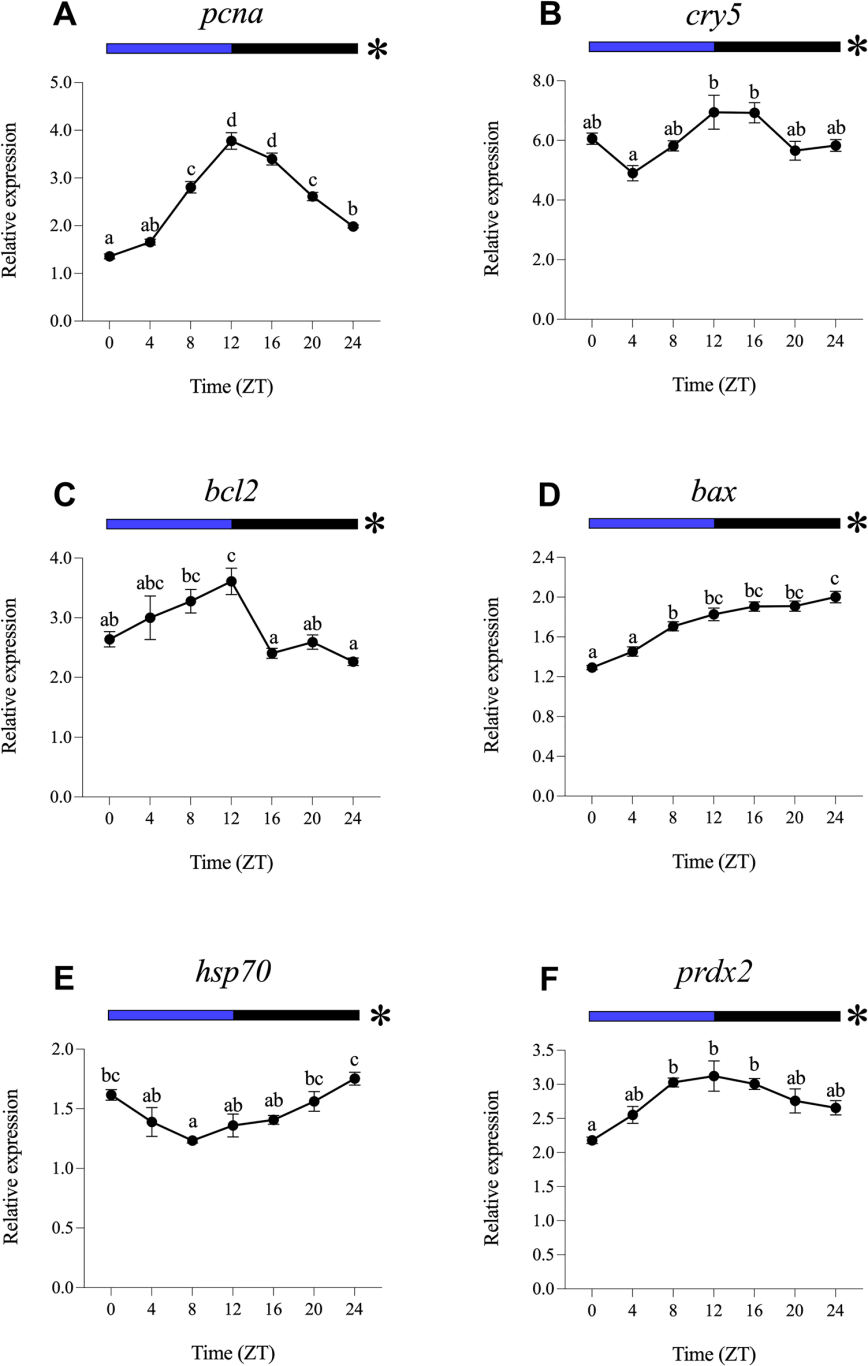
Relative daily gene expression of markers of cell proliferation (*pcna*, **A**), DNA repair (*cry5*, **B**), cellular apoptosis (*bcl2*, **C** and *bax*, **D**), cellular stress (*hsp70*, **E**) and oxidative stress (*prdx2*, **F**) in SAEC-H7 cells maintained under 12 h blue light-12 h darkness photoperiod (LDB). The x-axis shows time (hours) in Zeitgeber time (ZT). Lights on was at ZT0 and lights off was at ZT12. Samples were collected every 4 h and relative expression was determined by using real-time quantitative PCR (RT-qPCR). Each value represents the mean ± SEM (*n* = 6). Horizontal bars at the top indicate light (blue) and dark (black) phases. Asterisks represent significant daily rhythms determined by cosinor analysis. Different letters on top of the dots indicate statistically significant differences between mean values determined by one-way ANOVA followed by Tukey’s post-hoc test (significance at *p* < 0.05 or higher)

**Fig. 4:**
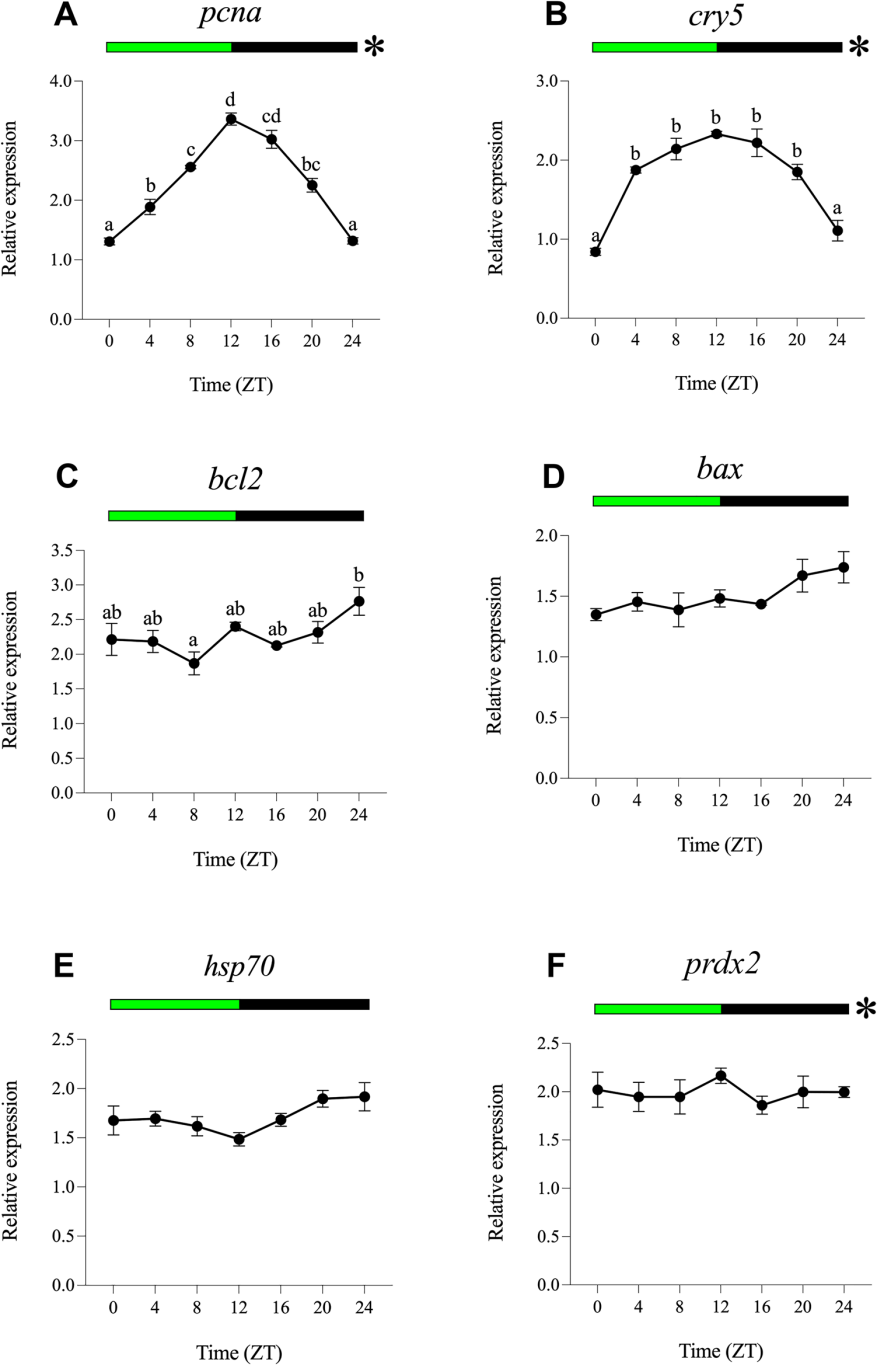
Relative daily gene expression of markers of cell proliferation (*pcna*, **A**), DNA repair (*cry5*, **B**), cellular apoptosis (*bcl2*, **C** and *bax*, **D**), cellular stress (*hsp70*, **E**) and oxidative stress (*prdx2*, **F**) in SAEC-H7 cells maintained under 12 h green light-12 h darkness photoperiod (LDG). The x-axis shows time (hours) in Zeitgeber time (ZT). Lights on was at ZT0 and lights off was at ZT12. Samples were collected every 4 h and relative expression was determined by using real-time quantitative PCR (RT-qPCR). Each value represents the mean ± SEM (*n* = 6). Horizontal bars at the top indicate light (green) and dark (black) phases. Asterisks represent significant daily rhythms determined by cosinor analysis. Different letters on top of the dots indicate statistically significant differences between mean values determined by one-way ANOVA followed by Tukey’s post-hoc test (significance at *p* < 0.05 or higher)

**Fig. 5:**
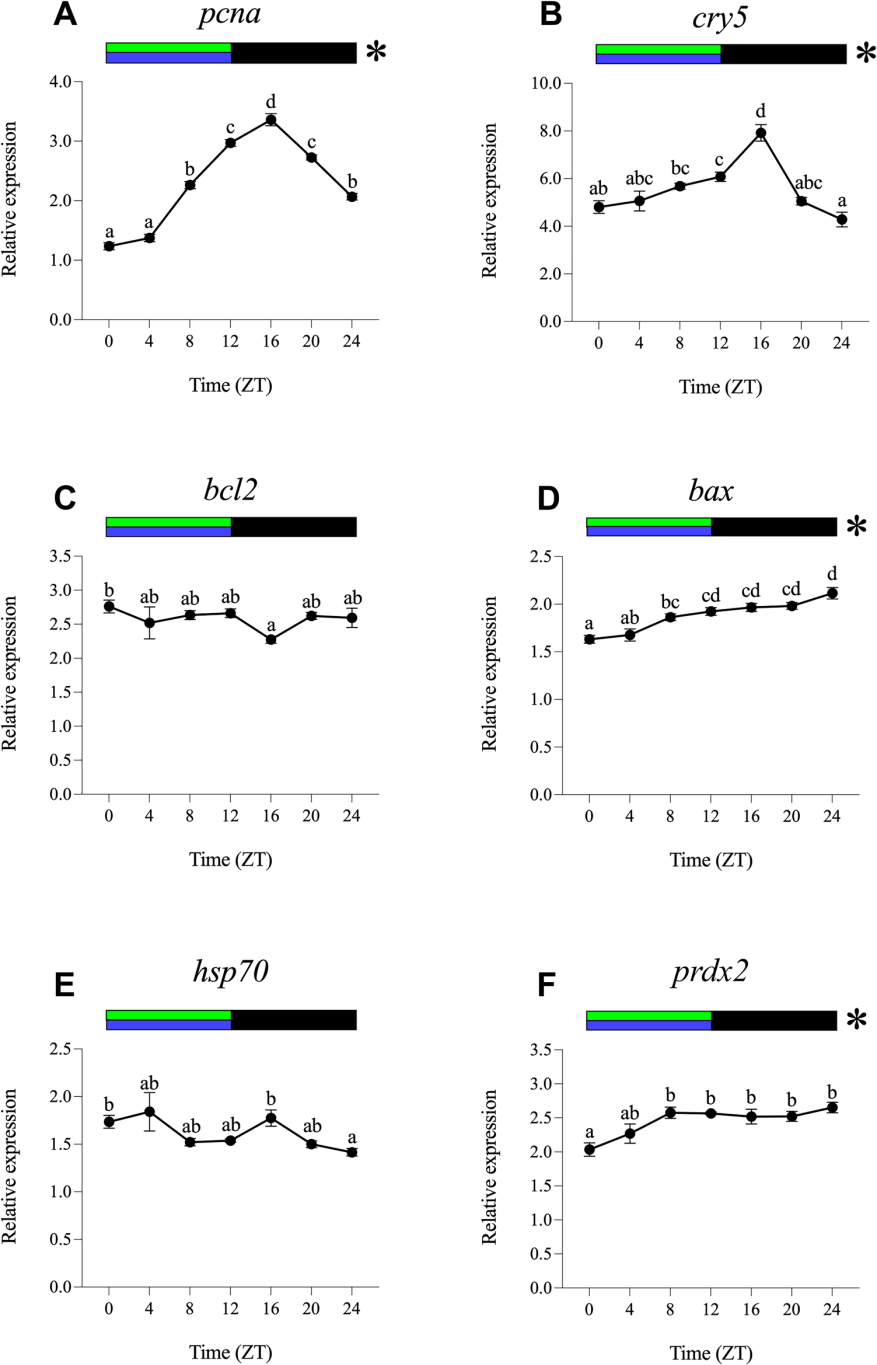
Relative daily gene expression of markers of cell proliferation (*pcna*, **A**), DNA repair (*cry5*, **B**), cellular apoptosis (*bcl2*, **C** and *bax*, **D**), cellular stress (*hsp70*, **E**) and oxidative stress (*prdx2*, **F**) in SAEC-H7 cells maintained under 12 h blue/green light-12 h darkness photoperiod (LDBG). The x-axis shows time (hours) in Zeitgeber time (ZT). Lights on was at ZT0 and lights off was at ZT12. Samples were collected every 4 h and relative expression was determined by using real-time quantitative PCR (RT-qPCR). Each value represents the mean ± SEM (*n* = 6). Horizontal bars at the top indicate light (blue/green) and dark (black) phases. Asterisks represent significant daily rhythms determined by cosinor analysis. Different letters on top of the dots indicate statistically significant differences between mean values determined by one-way ANOVA followed by Tukey’s post-hoc test (significance at *p* < 0.05 or higher)

**Fig. 6:**
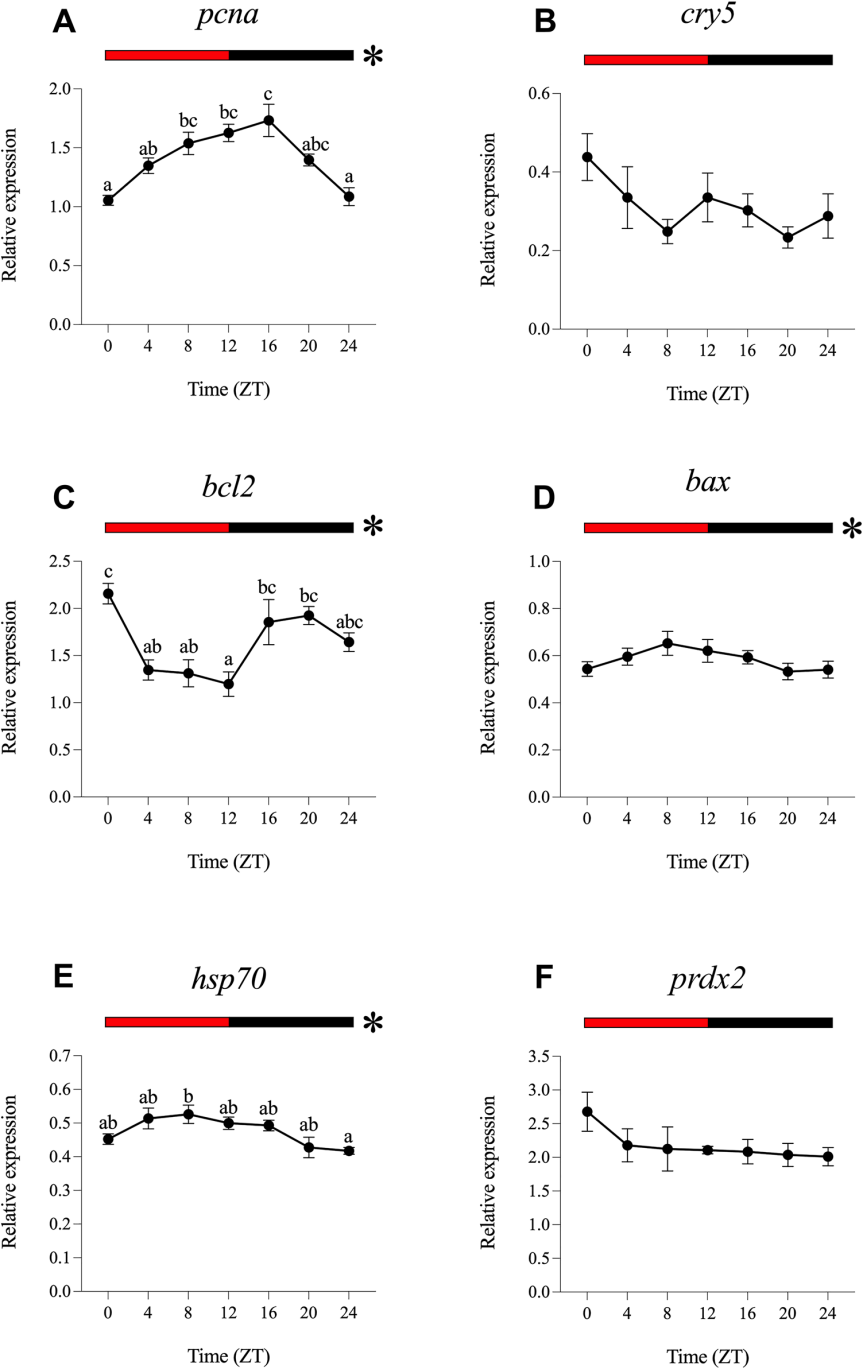
Relative daily gene expression of markers of cell proliferation (*pcna*, **A**), DNA repair (*cry5*, **B**), cellular apoptosis (*bcl2*, **C** and *bax*, **D**), cellular stress (*hsp70*, **E**) and oxidative stress (*prdx2*, **F**) in SAEC-H7 cells maintained under 12 h red light-12 h darkness photoperiod (LDR). The x-axis shows time (hours) in Zeitgeber time (ZT). Lights on was at ZT0 and lights off was at ZT12. Samples were collected every 4 h and relative expression was determined by using real-time quantitative PCR (RT-qPCR). Each value represents the mean ± SEM (*n* = 6). Horizontal bars at the top indicate light (red) and dark (black) phases. Asterisks represent significant daily rhythms determined by cosinor analysis. Different letters on top of the dots indicate statistically significant differences between mean values determined by one-way ANOVA followed by Tukey’s post-hoc test (significance at *p* < 0.05 or higher)

**Fig. 7:**
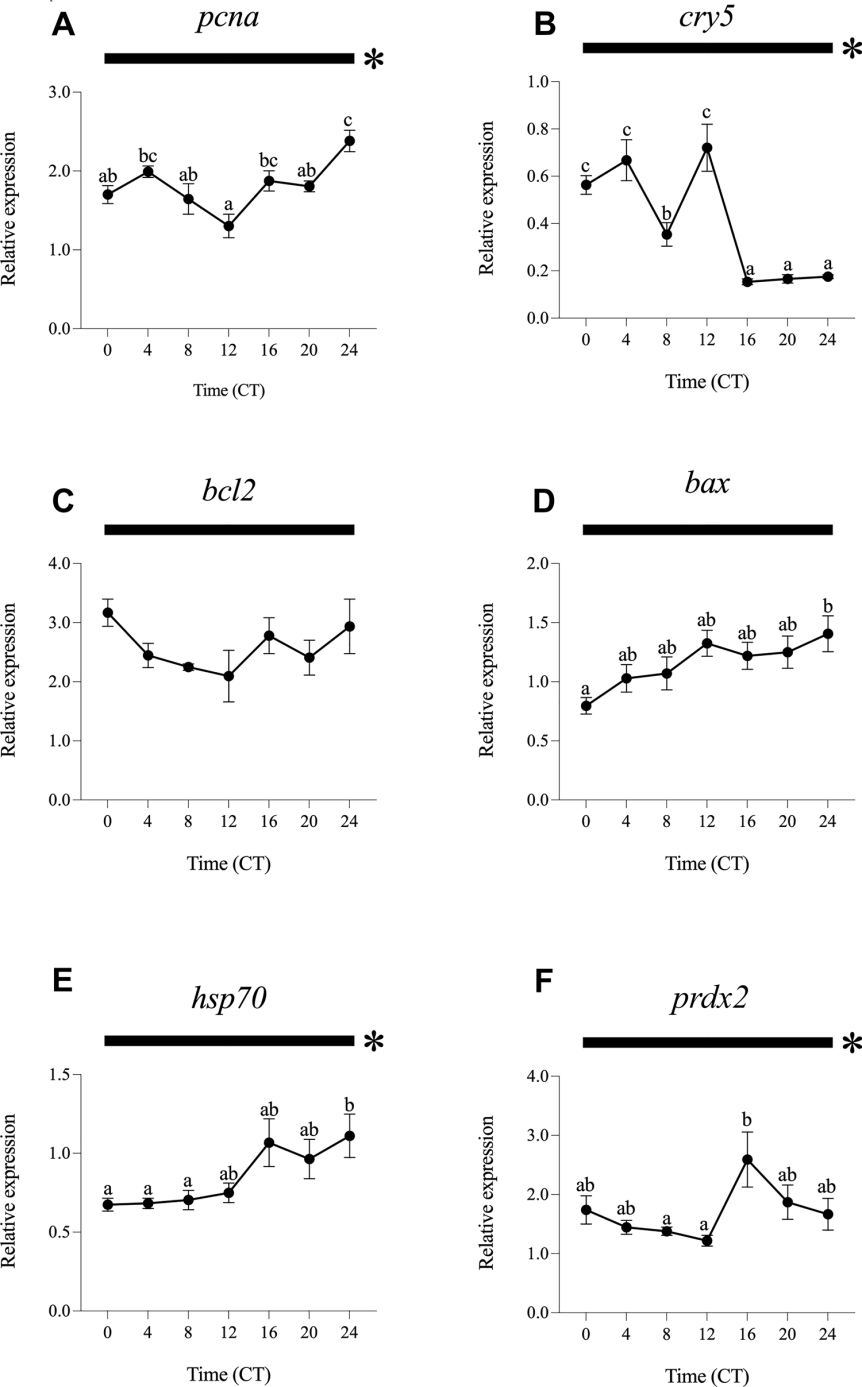
Relative daily gene expression of markers of cell proliferation (*pcna*, **A**), DNA repair (*cry5*, **B**), cellular apoptosis (*bcl2*, **C** and *bax*, **D**), cellular stress (*hsp70*, **E**) and oxidative stress (*prdx2*, **F**) in SAEC-H7 cells maintained under 24 h constant darkness (DD). The x-axis shows time (hours) in circadian time (CT). Samples were collected every 4 h and relative expression was determined by using real-time quantitative PCR (RT-qPCR). Each value represents the mean ± SEM (*n* = 6). Black horizontal bars at the top indicate 24-h constant dark conditions. Asterisks represent significant daily rhythms determined by cosinor analysis. Different letters on top of the dots indicate statistically significant daily differences between mean values determined by one-way ANOVA followed by Tukey’s post-hoc test (significance at *p* < 0.05 or higher)

For the analysis of daily gene expression and rhythms, data obtained under different experimental light conditions were normalised to ZT0 of the LDW group. The results obtained under the different light regimes (LDW, LDB, LDG, LDBG, LDR and DD) are represented in Figs. 2, 3, 4, 5, 6 and 7.

#### Daily Gene Expression Under White Light (LDW)

**Fig. 8:**
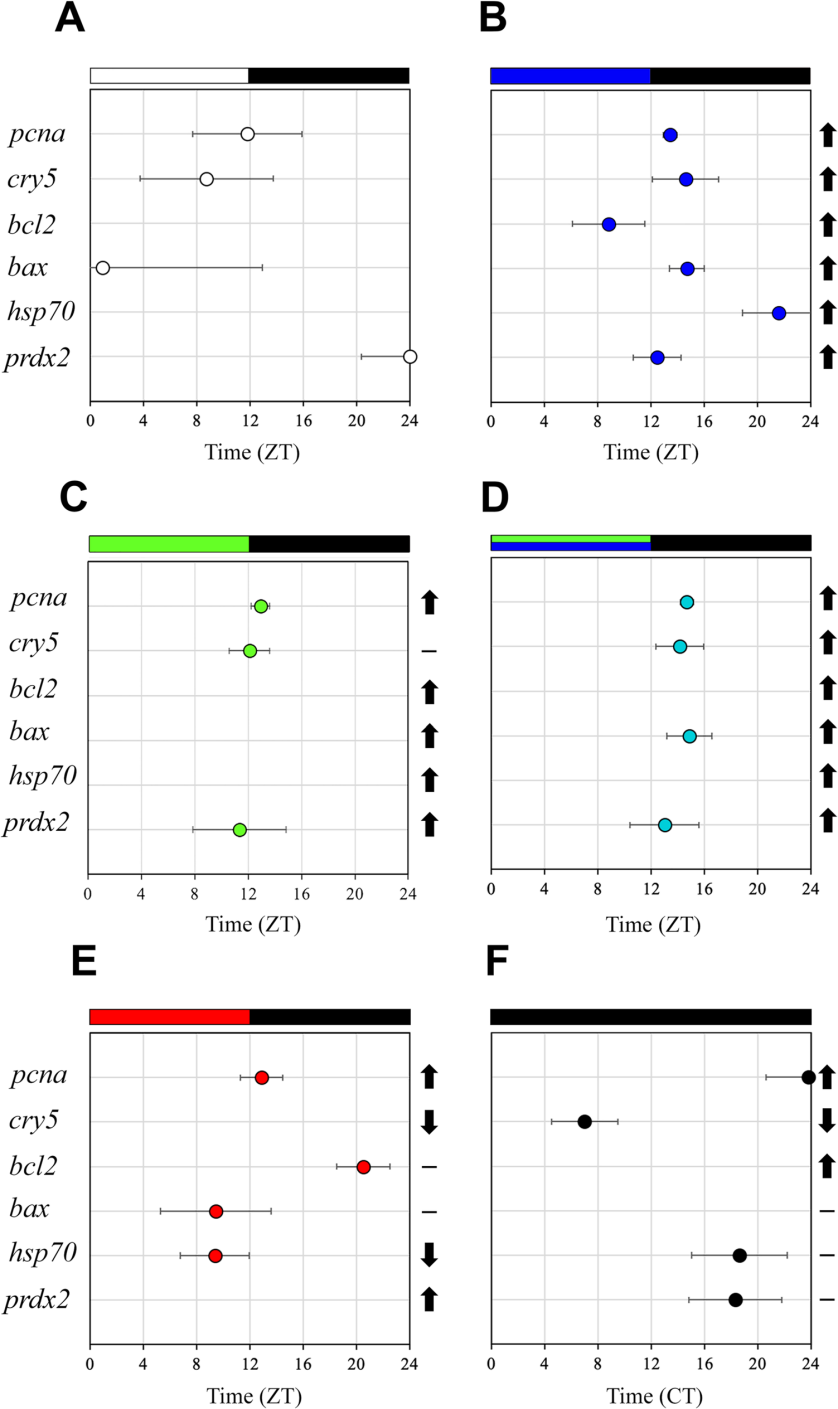
Map of acrophases of mRNA levels of genes involved in cell proliferation (*pcna*), DNA repair (*cry5*), cellular apoptosis (*bcl2* and *bax*), cellular stress (*hsp70*) and oxidative stress (*prdx2*) under the different photoperiod and light spectrum conditions analysed. **A** 12 h white light-12 h darkness photoperiod (LDW). **B** 12 h blue light-12 h darkness photoperiod (LDB). **C** 12 h green light-12 h darkness photoperiod (LDG). **D** 12 h blue/green light-12 h darkness photoperiod (LDBG). **E** 12 h red light-12 h darkness photoperiod (LDR). **F** 24 h constant darkness (DD); the acrophase (mean ± SEM) is indicated only for statistically significant rhythms (Cosinor, *p* < 0.05). The name of each target gene is indicated to the left. The white/coloured and dark bars above the panels (**A**–**E**) represent light–dark cycles of different wavelengths. The dark bar above the panel F represents the 24-h constant dark conditions. The time scale (x-axis) in **A**–**E** is expressed as Zeitgeber time (ZT), in which ZT0 corresponds to light onset and ZT12 to the switching off. The time scale (x-axis) in **F** is expressed as Circadian time (CT). On the right side of each panel is a summary of the expression of each gene under the different light conditions compared to LDW, which was used as a control condition (calibrator). ↑, significant increase; ↓, significant decrease; -, no differences

The one-way ANOVA revealed statistically significant daily differences in the transcript levels of *pcna*, *bax* and *prdx2* (Fig. 2A, D, and F). The daily expression of *pcna* showed its peak at the day-night transition (ZT12, Fig. 2A), whereas *bax* and *prdx2* exhibited antiphase daily expression patterns, peaking at the night-day transition (ZT0/24, Fig. 2D, F). The cosinor analysis showed significant daily rhythms of *pcna*, *cry5*, *bax* and *prdx2*, with acrophases at ZT11.48, ZT8.44, ZT0.55 and ZT23.59, respectively (Figs. 2A, B, D, F, 8A; Table 2). No daily differences or rhythms were observed for *bcl2* and *hsp70* (Figs. 2C, E, 8A; Table 2).

**Table 2:** Cosinor analysis of *proliferating cell nuclear antigen* (*pcna*), *cryptochrome-5* (*cry5*), *b-cell lymphoma-2* (*bcl2*), *bcl-2- associated X protein* (*bax*), *heat-shock protein-70* (*hsp70*) and *peroxiredoxin-2* (*prdx2*) daily relative expression in SAEC-H7 cells maintained under six different photoperiod and light spectrum regimes (LDW, LDB, LDG, LDBG, LDR and DD)

Light condition	Gene	Mesor (r.e.)	Amplitude (r.e.)	Acrophase (ZT/CT)	Significance (*p-value*)
LDW	*pcna*	0.99	0.17	11.48	*
	*cry5*	1.28	0.33	8.44	*
	*bcl2*	-	-	-	n.s
	*bax*	0.87	0.32	0.55	*
	*hsp70*	-	-	-	n.s
	*prdx2*	0.89	0.30	23.59	*
LDB	*pcna*	2.60	1.21	13.26	****
	*cry5*	6.05	0.85	14.36	**
	*bcl2*	2.92	0.50	8.49	**
	*bax*	1.68	0.29	14.43	****
	*hsp70*	1.43	0.17	21.35	**
	*prdx2*	2.77	0.44	12.27	***
LDG	*pcna*	2.40	0.96	12.56	****
	*cry5*	1.88	0.60	12.06	****
	*bcl2*	-	-	-	n.s
	*bax*	-	-	-	n.s
	*hsp70*	-	-	-	n.s
	*prdx2*	1.99	0.03	11.34	*
LDBG	*pcna*	2.32	1.09	14.41	****
	*cry5*	5.77	1.19	14.10	****
	*bcl2*	-	-	-	n.s
	*bax*	1.84	0.17	14.52	****
	*hsp70*	-	-	-	n.s
	*prdx2*	2.43	0.26	13.00	**
LDR	*pcna*	1.44	0.30	12.53	****
	*cry5*	-	-	-	n.s
	*bcl2*	1.59	0.41	20.32	****
	*bax*	0.59	0.06	9.27	*
	*hsp70*	0.48	0.05	9.23	***
	*prdx2*	-	-	-	n.s
DD	*pcna*	1.77	0.29	23.49	**
	*cry5*	0.39	0.21	7.00	*
	*bcl2*	-	-	-	n.s
	*bax*	-	-	-	n.s
	*hsp70*	0.85	0.19	18.39	*
	*prdx2*	1.69	0.48	18.19	*

SAEC-H7 cells were maintained for 2 days in each light condition and analysed on the third day. Rhythm parameters (mesor, amplitude, acrophase and significance) are indicated. Mesor and amplitude are given as relative expression (r.e.) values and acrophase as zeitgeber (ZT)/circadian (CT) times calculated by the cosinor method. Significance: *p < 0.05; **p < 0.001; ***p < 0.0001; ****p < 0.00001. n.s., non-significant rhythm. Abbreviations: LDW: 12 h white light-12 h darkness; LDB: 12 h blue light-12 h darkness; LDG: 12 h green light-12 h darkness; LDBG: 12 h blue/green light-12 h darkness; LDR: 12 h red light-12 h darkness; DD: constant darkness

#### Daily Gene Expression Under Blue Light (LDB)

All genes analysed displayed significant daily differences and daily rhythms under LDB, and with higher significance compared to the LDW condition, as revealed by one-way ANOVA and cosinor analyses (Fig. 3; Table 2). The daily profiles of *pcna*, *cry5*, *bcl2* and *prdx2* show their peaks in transcript levels close to the day-night transition (ZT12, Fig. 3A, B, C and F; Table 2). On the contrary, *bax* and *hsp70* exhibited their maximum expression peak at the night-day transition (ZT24, Fig. 3D and E; Table 2). Highly robust rhythms were revealed by cosinor, with acrophases at ZT13.26 for *pcna*, ZT14.36 for *cry5*, ZT8.49 for *bcl2*, ZT14.43 for *bax*, ZT21.35 for *hsp70* and ZT12. 27 for *prdx2* (Fig. 8B; Table 2). All the genes analysed exhibited higher mesors under LDB compared to their rhythmic counterparts in LDW condition, being also higher the amplitude of the rhythms in the case of *pcna* and *cry5* (Table 2).

#### Daily Gene Expression Under Green Light (LDG)

Green light leads to statistically significant daily variations in *pcna*, *cry5* and *bcl2* gene expression (Fig. 4A, B and C) but not in *bax*, *hsp70* or *prdx2* (Fig. 4D, E and F). In this case, the expression peaks of *pcna* and *cry5* (ZT12) were in antiphase with that of *bcl2* (ZT24). Cosinor analysis revealed robust rhythmic oscillation of *pcna* and *cry5* and significant but less evident daily rhythms of *prdx2*, with acrophases at ZT12.56, ZT12.06 and ZT11.34, respectively (Figs. 4A, B, F, 8C; Table 2). In general, mesors and amplitudes of these rhythms were higher under LDG than in LDW but lower compared to LDB condition (Table 2). No significant daily rhythms were detected for *bcl2*, *bax* or *hsp70* (Figs. 4C, D, and E, 8C; Table 2).

#### Daily Gene Expression Under Blue/Green Light (LDBG)

As in the case of LDB, all the genes analysed exhibited statistically significant daily variations under LDBG (Fig. 5). Transcript levels of *pcna* and *cry5* peaked in the first half of the night (ZT16, Fig. 5A and B), *bax* and *prdx2* expression increased more steadily at night (Fig. 5D and F), while *bcl2* and *hsp70* showed no clear daily expression pattern (Fig. 5C and E). Cosinor analysis revealed robust and significant daily rhythms for *pcna*, *cry5*, *bax* and *prdx2* under LDBG (Figs. 5A, B, D and F, 8D; Table 2). It is important to note that, with the exception of the *bax* gene, the acrophases, mesors and amplitudes of the daily rhythms under LDBG share similarities with those observed under LDB and LDG conditions (Fig. 8D; Table 2).

#### Daily Gene Expression Under Red Light (LDR)

Red light determines statistically significant daily variations in three of the six genes analysed, i.e., *pcna*, *bcl2* and *hsp70,* but with markedly different daily profiles (Fig. 6A, C and E). Thus, *pcna* exhibited higher transcript levels at the day-night transition, peaking at ZT16 (Fig. 6A), *bcl2* showed higher nocturnal expression, with a peak at ZT0 (Fig. 6C), while *hsp70* displayed the highest mRNA levels during the day, with maximum values at ZT8 (Fig. 6E). On the other hand, LDR leads to statistically significant rhythmic oscillations in the same genes, as well as in *bax*, as revealed by cosinor (Figs. 6A, C, D and E, 8E; Table 2). The acrophases of the daily rhythms of *pcna*, *bcl2*, *bax* and *hsp70* were located at ZT12.53, ZT20.32, ZT9.27 and ZT9.23, respectively (Fig. 8E; Table 2), being most of the mesors and amplitudes of the rhythms lower when compared to LDB (Table 2).

#### Daily Gene Expression Under Constant Darkness (DD)

SAEC-H7 cells maintained in constant darkness for 3 days still showed statistical differences in the daily expression of *pcna*, *cry5*, *bax*, *hsp70* and *prdx2* on day 3 (Fig. 7A, B, D, E and F). Under DD, *pcna*, *bax* and *hsp70* peaked at the end of the subjective night/onset of the subjective day (CT24) while *cry5* and *prdx2* peaked at CT12 and CT16, respectively (Fig. 7A, B, D, E and F). Cosinor analysis revealed significant circadian rhythms of *pcna*, *cry5*, *hsp70* and *prdx2*, in most cases with lower significance compared to their counterpart rhythmic genes at LDB, LDG, LDBG and LDR conditions (Table 2). The acrophases of these circadian rhythms were located at CT23.49 for *pcna*, CT7.00 for *cry5*, CT18.39 for *hsp70* and CT18.19 for *prdx2* (Fig. 8F; Table 2). All the rhythmic genes analysed exhibited lower mesors under DD compared to the LDB condition, being also lower the amplitude of the rhythms in the case of *pcna* and *cry5* (Table 2). No significant daily variation or rhythm was detected for *bcl2* under DD (Figs. 7C and 8F; Table 2).

## Discussion

In the present work, we have shown marked effects of photoperiod and light spectrum on the daily gene expression and rhythms of cellular markers of proliferation (*pcna*), DNA repair (*cry5*), apoptosis (*bcl2*, *bax*) and cellular/oxidative stress (*hsp70*, *prdx2*) using an embryonic stem cell line (SAEC-H7) obtained from a marine teleost fish, the gilthead seabream. The highest expression levels and most robust daily differences and/or rhythms of genes analysed were observed under blue (LDB) and blue/green (LDBG) lights (Fig. 8). Under LDB condition, peaks in transcript levels of cell proliferation (*pcna*), DNA repair (*cry5*), anti-apoptotic (*bcl2*) and oxidative stress (*prdx2*) markers were in antiphase with those of pro-apoptotic (*bax*) and cell stress (*hsp70*) markers (ZT12 in the former *versus* ZT24 in the latter). Moreover, four of the six genes analysed (*pcna*, *cry5*, *hsp70* and *prdx2*) exhibited significant rhythms after 3 days in constant darkness (DD), supporting the endogenous and circadian nature of these rhythms (Fig. 8). However, most of these circadian rhythms under DD showed affected mesors, amplitudes and/or phases when compared to those found under light–dark cycles of different spectra. Interestingly, *bax* expression showed significant daily differences (ANOVA) but no significant daily rhythms (cosinor) under DD. Monitoring *bax* gene expression for more than one daily cycle in DD could clarify whether this gene definitively does not show circadian rhythms or whether there is an endogenous rhythm with a period longer than 24 h. These results, which reveal the spectral sensitivity of embryonic cells of gilthead seabream at least as early as the morula stage, reinforce and extend our previous studies, showing that SAEC-H7 cells were highly sensitive to light and exhibited a robust and endogenous molecular clock machinery (Vergès-Castillo et al. 2021, 2024). In a previous study performed using the PAC2 zebrafish embryo-derived cell line, it was shown that light-inducible clock genes (*per2*, *cry1a*, *cry5*) are differentially activated by blue and red light via the MAPK signalling pathway and the D-box enhancer promoter element (Mracek et al. 2013). Therefore, spectral sensitivity in light-induced gene expression appears to be a common feature of embryonic cell lines from both freshwater and marine fish.

Light sensitivity has been observed from very early stages of development in other fish species (Davie et al. 2011; Dekens and Whitmore 2008; Martín-Robles et al. 2012). Moreover, fish embryo and cell lines derived from early-stage embryo and larvae have shown broad spectral sensitivity, as well as the expression a number of opsin photopigments, several of which are under direct clock control (Farhat et al. 2009; Frau et al. 2020a, 2022; Steindal and Whitmore 2020). Supporting these evidences, light spectrum and photoperiod during incubation and early life affect hatching, development, growth, malformations, survival, and/or feeding and locomotor behaviour in fish species as Senegalese sole, *Solea senegalensis* (Blanco-Vives et al. 2010, 2011, 2012), European sea bass, *Dicentrarchus labrax* (Villamizar et al. 2009), zebrafish (Villamizar et al. 2014; de Alba et al. 2022), Atlantic cod, *Gadus morhua,* and turbot, *Scophthalmus maximus* (Sierra-Flores et al. 2016), with light/dark cycles of blue wavelengths providing the best results in terms of performance.

Proliferating cellular nuclear antigen (PCNA) is a eukaryotic replicative DNA clamp that plays multiple roles in chromosome duplication and repair of damaged DNA (Kang et al. 2024). PCNA is a key cell cycle regulator for the G1/S transition and DNA synthesis during replication (Bravo et al. 1987) and the regulation of PCNA cycling during DNA synthesis is critical for genome integrity (Kang et al. 2024). Transcription of *pcna* in SAEC-H7 cells showed daily differences and rhythms under all photoregimes tested, with maximum levels at the day-night transition in all light spectra, which is consistent with the daily expression pattern observed in our previous study (Vergès-Castillo et al. 2021). Moreover, the maintaining of significant *pcna* rhythms after 3 days in constant light (Vergès-Castillo et al. 2021) and constant dark (present study) conditions evidences that this cell cycle regulator is under strong control of the molecular clock. Similar results implicating the circadian clock in the mediation of the effects of light on the cell cycle were reported in zebrafish larvae and embryo-derived cell lines (Dekens et al. 2003). Interestingly, *pcna* expression in SAEC-H7 cells is in phase with *clock* and *bmal1* transcript levels reported in our previous study (Vergès-Castillo et al. 2024), suggesting that Clock/Bmal1 complex might mediate the rhythmic transcriptional activation of *pcna* gene. In the retina of the teleost *Astatotilapia burtoni*, the diurnal rhythmic expressions of both *clock/bmal* and *pcna* genes are also light-dependent (Song et al. 2017). As the *pcna* promoter region of this species exhibits five E-boxes, which could represent regulatory sites for Clock protein, it has been proposed that Clock may contribute to the rhythmic proliferation of retinal progenitor cells through *pcna* (Song et al. 2017). Whether similar regulatory mechanisms exist in SAEC-H7 cells should be elucidated in future studies.

The timing of cell proliferation is critical for the development and survival of the organisms (Johnson 2010), and this is particularly important for fish, which in most cases exhibit floating eggs and larvae exposed to highly mutagenic UV radiation from sunlight. Therefore, a greater proliferation rate at the end of the day/beginning of the night could represent an advantage to avoid UV radiation and minimise DNA damage in highly proliferating periods of development. Although we lack data on Pcna protein levels, our gene expression results suggest that not only the light–dark cycle but also the light spectrum may be important for the synchronisation of cell proliferation and cell cycle progression in SAEC-H7 cells because the overall expression of *pcna* and mesors and the amplitudes of their daily rhythms were higher under LDB, LDG and LDBG compared to LDW and LDR. Neural stem/progenitor cells of mice constitutionally expressed opsins responsive to blue/red lights, and blue light was more effective at triggering their proliferation and self-renewal than red light (Wang et al. 2019). It has been also shown that blue LED lights induced pancreatic β cell proliferation in mice (Kawana et al. 2024). However, irradiation with blue LED light inhibited the proliferation of mesenchymal stem cells from human (Zhu et al. 2019b) and mouse (Yuan et al. 2017) origins and reduced the proliferation of human dermal fibroblasts in a dose- and wavelength-dependent manner (Opländer et al. 2011), whereas red light accelerated epidermal proliferation in both an epidermal-equivalent model and human skin (Umino and Denda 2023). In addition, the effect of blue light on the healing process has also been investigated, showing variable actions on proliferation depending on the energy of the light source (Prado et al. 2023). Further studies taking into account not only wavelength but also light intensity should therefore be carried out, particularly in fish, to better define the nature of the actions of lights of different spectra on cell proliferation.

On the other hand, we analysed the effect of photoperiod and light spectrum on the expression of *cryptochrome 5* (*cry5*) or *6–4 photolyase*. Cryptochromes are members of the DNA photolyase/cryptochrome flavoprotein family and regulate some of the blue light responses in plants, such as growth and development, synchronise the circadian rhythm with the daily light–dark cycle and have been shown to function as light-inducible DNA repair enzymes in animals (Sancar 2003; Tamai et al. 2004). Our results showed the highest *cry5* transcript levels under LDB and LDBG, and the lowest expression of this DNA repair enzyme (ten- to 12-fold lower) in LDR and DD conditions. These results are consistent with those reported in the PAC2 zebrafish embryo-derived cell line, where blue light induces *cry5* gene expression, resulting in significantly higher transcript levels after 6 h of light exposure compared to DD or red light (Mracek et al. 2013). Except for LDR, significant daily variations and/or rhythms of *cry5* were observed in all photoregimes (including DD), showing their peaks/acrophases from the second half of the day to the beginning of the night, thus resembling those observed for *pcna*. A very marked daily spawning rhythm has been reported in gilthead seabream, which start spawning in the afternoon and reach its peak before dusk, in anticipation of the night (Meseguer et al. 2008). In this way, the highest *cry5* expression under LDB and LDBG in embryonic cells could contribute to increasing Cry5 protein levels, reducing damage and conferring an advantage at early proliferative stages, when embryos are undergoing intense DNA replication and rapid mitosis (Tamai et al. 2004). This could explain why in several fish species light/dark cycles of blue wavelength provide the best results in terms of survival, growth, and development, while red light and continuous dark conditions lead to high malformation and mortality rates in the early embryonic and larval stages (Blanco-Vives et al. 2010, 2011, 2012; de Alba et al. 2022; Frau et al. 2020a; Sierra-Flores et al. 2016; Villamizar et al. 2009, 2011, 2014). Alternatively, it could be argued that LDB and LDBG cause more cell damage, explaining why *cry5* expression is stimulated under these conditions. However, if this were the case, one would expect higher mortality under these light regimes, which is not the case, as reported above. Furthermore, LDB and LDBG stimulate the expression not only of *cry5* but also of the other genes analysed, suggesting that the increase in *cry5* expression is the result of a generalised induction of gene transcription under these light conditions.

Apoptosis is a genetically programmed cell death that is particularly active during development, ageing and different pathologies, being its balance with cell proliferation necessary for maintaining homeostasis in healthy tissues (Danial and Korsmeyer 2004). B-cell lymphoma-2 (BCL-2) family proteins regulate this programmed cell death, with some members of the family (such as BCL-2 and BCL-XL) being inhibitors of apoptosis, whereas others (such as BAX and BAK) promote cell death (Youle and Strasser 2008). BCL-2 is located in the outer mitochondrial membrane where it can bind and sequester pro-apoptotic activator proteins such as BIM, PUMA or truncated BID, thus acting as a pro-survival protein. In turn, BAX oligomerises and forms pores to cause permeabilisation of the outer mitochondrial membrane, resulting in the release of several molecules from the mitochondrial intermembrane space, including second mitochondria-derived activator of caspases, serine proteases and cytochrome c, which initiate the apoptotic process (Singh et al. 2019). In SAEC-H7 cells, we have identified conspicuous effects of light spectrum on gene expression of anti-apoptotic *bcl2* and pro-apoptotic *bax*, with higher transcript levels of both genes under LDB, LDG and LDBG and lower expression under LDW and LDR. Daily differences and rhythms were also affected by light spectrum, being only significant for both *bcl2* and *bax* under LDB. In this wavelength regime, the acrophase of *bcl2* rhythm (ZT8.49) precedes 5 h this of *pcna* (ZT13.26), whereas *bax* expression increases gradually during the night, coinciding with the decline in *pcna* transcript levels. This could reflect the segregation of the transcription of genes involved in cell proliferation and apoptosis at certain times of the day under LDB, when conditions are optimal for each process. However, both transcriptional activities appear to be desynchronised in LDR, with the acrophase of pro-apoptotic *bax* occurring in the second half of the day (ZT9.27), anticipating that of *pcna* (located at ZT12.53, in the transition between the day and night), while the acrophase of the anti-apoptotic *bcl2* is displaced to the end of the night (ZT20.32). Kim and co-workers have investigated how irradiation of specific wavelengths (white, blue, green, red) regulated apoptosis in heat-stress-exposed olive flounder, *Paralichthys olivaceus* (Kim et al. 2016). In this species, light with short wavelengths (blue and, particularly, green light), effectively reduced the warming-induced apoptosis, whereas red light increased apoptosis (Kim et al. 2016). In contrast, blue LED light-induced cell damage and apoptosis was observed in goldfish retina as revealed by the induction of caspase 3 and TUNEL assay (Song and Choi 2019). Whether and how altered daily patterns and rhythms of Bcl-2 family proteins and other markers of apoptosis/proliferation (e.g., Caspases, Pcna) mediate the effects of the light spectrum on the development of gilthead seabream and other fish species in vivo needs to be further investigated in the future using DNA fragmentation and BrdU assays.

Heat shock proteins (HSPs) are a class of highly conserved proteins that are synthesised in organisms in response to environmental stress, enhancing cellular resistance to extreme conditions, with the *hsp70* gene family being one of those that have attracted the most attention in fish (Chen et al. 2024). In turn, Peroxiredoxin (PRDX) is a family of cellular antioxidant enzymes that protect organisms from oxidative stress caused by reactive oxygen species (ROS), play an important role in immune cytotoxicity, act as modulators of inflammation, protect against cell death and tumour progression, and facilitate tissue repair after damage (Valero et al. 2015; Zhu et al. 2019a). Both *hsp70* and *prdx2* have also been associated with response to light-induced cellular and oxidative stress (Kim et al. 2014; Tanito et al. 2008; Wu et al. 2019). In the present study, a higher expression of *hsp70* was found under LDB, LDG and LDBG compared to LDW, LDR and DD. In the case of *prdx2*, similar results were observed but its transcript levels were also high in LDR. The higher expression of both *hsp70* and *prdx2* in LDB, LDG and LDBG suggests that SAEC-H7 cells may have an increased ability to cope with external stressors when subjected to a potentially adverse stimulus. If a similar induction of *hsp70* and *prdx2* transcript levels is sustaining the best performance of embryo and larvae of Senegalese sole (Blanco-Vives et al. 2010, 2011, 2012), European sea bass (Villamizar et al. 2009), zebrafish (Villamizar et al. 2014; de Alba et al. 2022), Atlantic cod and turbot (Sierra-Flores et al. 2016) under LDB photoregime remains to be elucidated. High levels of *hsp70* expression have also been reported in turbot larvae exposed to LDB and LDG (Wu et al. 2019) and *Hsp70* expression and ROS generation in morula of hamsters (*Mesocricetus auratus*) were increased in the blue light but decreased in the red lighting compared with the visible (white) light (Oh et al. 2007). We also observed daily differences and/or rhythms of *hsp70* (LDB, LDBG, LDR, DD) and *prdx2* (LDW, LDB, LDG, LDBG, DD) transcript levels under particular photoregimes. The maintaining of *hsp70* and *prdx2* rhythms after 3 days in DD reflects the endogenous nature of these rhythms in SAEC-H7 cells. Studies performed in killifish (*Fundulus heteroclitus*) and zebrafish found daily patterns of *hsp70* mRNA levels with higher values appearing at midday (de Alba et al. 2021; Healy and Schulte 2012). However, in our embryonic cells, peaks of expression were dependent on the light regime, being placed at the end of the night/beginning of the day under LDB and LDBG, in the second half of the day in LDR and in the middle of the subjective night in DD. The oxidation state of highly conserved peroxiredoxin (PRDX) proteins exhibits circadian oscillations in cells from humans, mice, zebrafish and marine algae, probably reflecting an endogenous rhythm in the generation of reactive oxygen species (Edgar et al. 2012; Sandbichler et al. 2018). Edgar and co-workers speculate that sensing and responding to oxidative cycles in cellular environments could have driven the evolution of circadian rhythms and maintained the intrinsic link between clocks and metabolism (Edgar et al. 2012). In this sense, it is interesting to note that in SAEC-H7 cells, acrophases of *hsp70* and *prdx2* circadian rhythms are on CT18.39 and CT18.19, respectively, after 3 days in DD, which are almost coincident with those reported for *clock* (CT17.57) and *bmal1* (CT18.34) after 3 days in constant darkness in our previous study (Vergès-Castillo et al. 2024).

In summary, we have shown here that SAEC-H7 cells are highly rhythmic and sensitive not only to the light–dark cycle but also to the light spectrum, as reported for the PAC2 zebrafish embryo-derived cell line (Mracek et al. 2013), making this marine embryonic cell line a very useful tool for circadian and environmental biology studies. Our results have shown for the first time in marine fish how photoperiod and light spectrum can affect the daily gene expression and rhythms of cellular markers of proliferation, DNA repair, apoptosis and cellular/oxidative stress. We can therefore assume that not only light–dark cycles (Dekens et al. 2003) but also light wavelengths may play a crucial role in modulating the induction and synchronisation of daily rhythms of genes critical for cell cycle progression. In any case, it should be kept in mind that the processes analysed are complex and multifactorial, and other molecular players interacting with the genes targeted in this study would deserve attention in future studies. Taking into account the importance that both photoperiod and light spectrum play in the early stages of fish, and considering the high commercial value and wide consumption of the gilthead seabream, the information obtained in this study could also be relevant to develop light protocols that improve the development, breeding and welfare of this species in the aquaculture industry. Furthermore, our results have allowed us to consolidate the utility of the developed embryonic stem cell line as a reliable biotechnological tool for in vitro studies, to complement in vivo animal experiments according to the 3Rs principle. These results also highlight the importance of implementing photoperiods with light–dark cycles of appropriate wavelengths (e.g., blue light) when conducting research on fish cell culture.

## Data Availability

Sequences are available in the NCBI databases. Data will be made available upon request.

## Abbreviations

*bax*: *bcl2-associated X protein*
*bcl2*: *b-cell lymphoma-2*
*cry5*: *cryptochrome-5 or 6–4 photolyase*
CT: Circadian time
DD: Constant darkness
*hsp70*: *heat-shock protein-70*
LDB: Blue light–dark cycle
LDBG: Combined blue/green light–dark cycle
LDG: Green light–dark cycle
LDR: Red light–dark cycle
LDW: White light–dark cycle
nm: Nanometres
*pcna*: *Proliferating cell nuclear antigen*
*prdx2*: *peroxiredoxin-2*
RT-qPCR: Real-time quantitative polymerase chain reaction
ZT: Zeitgeber time
